# A case of recurrent respiratory papillomatosis with malignant transformation, HPV11 DNAemia, high L1 antibody titre and a fatal papillary endocardial lesion

**DOI:** 10.1186/1743-422X-11-114

**Published:** 2014-06-18

**Authors:** Paul-Stefan Mauz, Manola Zago, Ralf Kurth, Michael Pawlita, Martin Holderried, John Thiericke, Angelika Iftner, Frank Stubenrauch, Karl Sotlar, Thomas Iftner

**Affiliations:** 1 Department of Otolaryngology, Head and Neck Surgery, University Hospital Tuebingen, Tuebingen, Germany; 2 Division of Experimental Virology, Institute for Medical Virology, University Hospital Tuebingen, Elfriede-Aulhorn-Str. 6, 72076 Tuebingen, Germany; 3 Institute of Pathology, University Hospital Tuebingen, Tuebingen, Germany; 4 Department of Genome Modifications and Carcinogenesis, Research Program Infection and Cancer, German Cancer Research Centre (DKFZ), Heidelberg, Germany; 5 Institute of Pathology, University of Munich, Munich, Germany

**Keywords:** JORRP, HPV11, HPV DNAemia

## Abstract

**Background:**

Recurrent respiratory papillomatosis (RRP) is a rare disease, which is characterised by the growth of papillomavirus-induced papillomas within the respiratory tract. Malignant transformation occurs in less than 1% of the cases.

**Case presentation:**

We report a case of human papillomavirus (HPV) type 11-associated juvenile-onset RRP (JORRP) initially diagnosed at the age of two years. Remarkably high copy numbers of HPV11 DNA and antibody titres targeting the capsid protein L1 were detected in the patient’s serum. The patient developed squamous cell carcinomas in both lungs and extraordinarily an HPV11 DNA-positive papillary endocardial lesion in the left atrium of the heart, which caused thromboembolic events leading to the patient’s death at 19 years old.

**Conclusion:**

We here report a severe case of JORRP hallmarked by HPV11 DNAemia and very high antibody titres directed against the major viral capsid protein L1. Furthermore, the extent of malignant transformation and the discovery of a very rare fatal endocardial lesion highlight the unpredictability of JORRP and the complexity of its clinical management.

## Background

Juvenile onset recurrent respiratory papillomatosis (JORRP) is a relatively rare disease, but still the most common pediatric neoplasm found in the larynx. Papillomas generally appear in the larynx of children before the age of five and recur after surgical excision in a majority of cases. Only 5% of the patients show involvement of trachea and bronchi and 1% of patients will develop manifestations within the lung parenchyma [1,2]. Rarely, spontaneous carcinomatous transformation occurs [3]. Generally, distant metastases do not occur [2]. Clinical and epidemiological studies have determined that HPV is the etiological agent of JORRP [4,5] with HPV6 and 11 accounting for most cases. Most reports of JORRP-associated squamous cell carcinomas of the lung, in which an HPV type was determined, describe male patients with HPV11-associated JORRP [3,6-11].

Here we describe a 19-year-old male patient with an aggressive form of JORRP that required a total of 132 interventions within the 17 years following diagnosis.

## Case presentation

A 14 year old male patient was referred to the Department of Otolaryngology, Head and Neck Surgery at the University Hospital Tuebingen in August 2002. Diagnosed at the age of two he underwent tracheostomy and since had been treated for juvenile onset recurrent respiratory papillomatosis (JORRP) through multiple surgeries. Papillomas were detectable in the larynx, trachea, and main bronchi and the patient had progressive lung manifestations*.* At the time of referral the patient had already undergone 116 operations. A total of 16 surgical excisions and laser ablations were performed during the following 5 years. In addition to ablative therapies, the patient was treated with the antiviral drug cidofovir for three years (2004–2007): first intralesionally, later systemically and finally via inhalations.

At the age of 18, he presented with a tracheal stenosis that was treated by balloon dilatation; he was also affected by pneumothorax and subcutaneous emphysema of his upper body, which was treated by a thoracic drainage. One year later the patient developed symptoms of Leriche’s syndrome caused by thromboembolic occlusion of the aortoiliac bifurcation. An embolectomy was performed. Additional thromboembolic events caused minor infarctions in both kidneys and the spleen. An ischemic stroke in the supply area of the left middle cerebral artery caused aphasia, hemiparesis and facial nerve paresis. Four days later, another thromboembolic crisis led to an occlusion of both femoral arteries. Magnetic resonance imaging revealed a suspicious mass in the left cardiac atrium, involving the right pulmonary vein. About one month later a series of thromboembolic events involving the brain, liver, heart, kidneys, aorta, and pelvic arteries occurred. The patient finally died and an autopsy was performed at the Institute of Pathology, University Hospital Tuebingen.

### Association of the patient’s disease with HPV11

To characterise the patient’s viral infection in more detail, DNA was extracted from surgically removed laryngeal papillomas. Exclusively HPV11 DNA was detected in the specimens and qRT-PCR (see Additional file 1) estimated the viral genome copy number as 1.2 × 10^4^ copies/cell. These viral load values are suggestive of a productive infection at the larynx. A full length HPV11 genome was isolated from a laryngeal papilloma and completely sequenced. The genome was 99% identical to the prototype sequence [12] (NCBI number: M14119), with a total of 27 nucleotide deviations. Fifteen of which did not result in amino acid substitutions in the respective proteins. Three mutations, one deletion and three nucleotide insertions occurred in the Long Control Region. The remaining five mutations affected the amino acid sequence of viral proteins (Table 1), however, the analysis of the protein sequences revealed that these mutations do not occur in conserved or functional domains and most probably do not interfere with the proteins’ activities.

**Table 1:** Detected amino acid changes within the isolated HPV11 genome

**Nucleotide position**	**Reference sequence HPV11**	**Detected change**	**Affected amino acid**	**Affected viral protein**
662	G	T	Ala 45 → Ser 45	E7
1783-1784	CG	GC	Arg 318 → Ala 318	E1
3645	A	G	Lys 308 → Arg 308	E2
3952	A	T	Leu 28 → Phe 28	E5A
3991	G	C	Val 41 → Leu 41	E5A

Patient’s blood samples were investigated for the presence of HPV11 (Table 2). Total DNA was extracted from 200 μl of whole blood, plasma, leukocyte and erythrocyte fractions. HPV11 DNA was only detected in whole blood and the plasma fraction but not in any of the cellular fractions. qRT-PCR determined that 8.85 × 10^5^ viral genome copies/ml were present in the whole blood and 1.55 × 10^6^ viral genome copies/ml were measured within the plasma fraction. These data suggest that most viral genomes were not cell associated, which was supported by the data obtained by measuring the viral load within the filtered plasma fraction (0.2 μm filters), where all possible residual cells were eliminated, and 5.61 × 10^5^ viral genome copies/ml remained. As a way of indirectly demonstrating the presence of viral particles, plasma samples were treated with Benzonase in order to digest all unencapsidated DNA. Benzonase treatment has previously been reported to be both effective and safe for the elimination of free viral DNA from papillomavirus suspensions [13]. Our results indicate that probably no viral particles were present within the patient’s plasma samples (Table 3).

**Table 2:** Viral genome copy numbers in whole blood and plasma

**Patient specimen**	**Viral genome copies/ml**
Whole blood	8.85 × 10^5^
Plasma fraction	1.55 × 10^6^
Filtered plasma fraction	5.61 × 10^5^

**Table 3:** Viral particles are not present in the patient’s plasma after treatment with Benzonase

	**Proteinase K**	**Benzonase**	**LiPa genotyping results**
SiHa control	+		HPV16
SiHa control	+	+	HPV-negative
SiHa control		+	HPV-negative
Plasma	+		HPV11
Plasma	+	+	HPV-negative
Plasma		+	HPV-negative

The patient’s immune-response against HPV was determined in blood samples from August 2005 and April 2007. In 2005 a significant response to the major capsid protein L1 of HPV11 was present with about 3,000 median fluorescence units (MFI) at a dilution of 1:100. The antibody response increased further to 30,000 MFI in the 2007 sample indicating a more than 10-fold increase in titre. No antibodies against HPV11 E6 and E7 were detected.

### Autopsy findings

**Figure 1:**
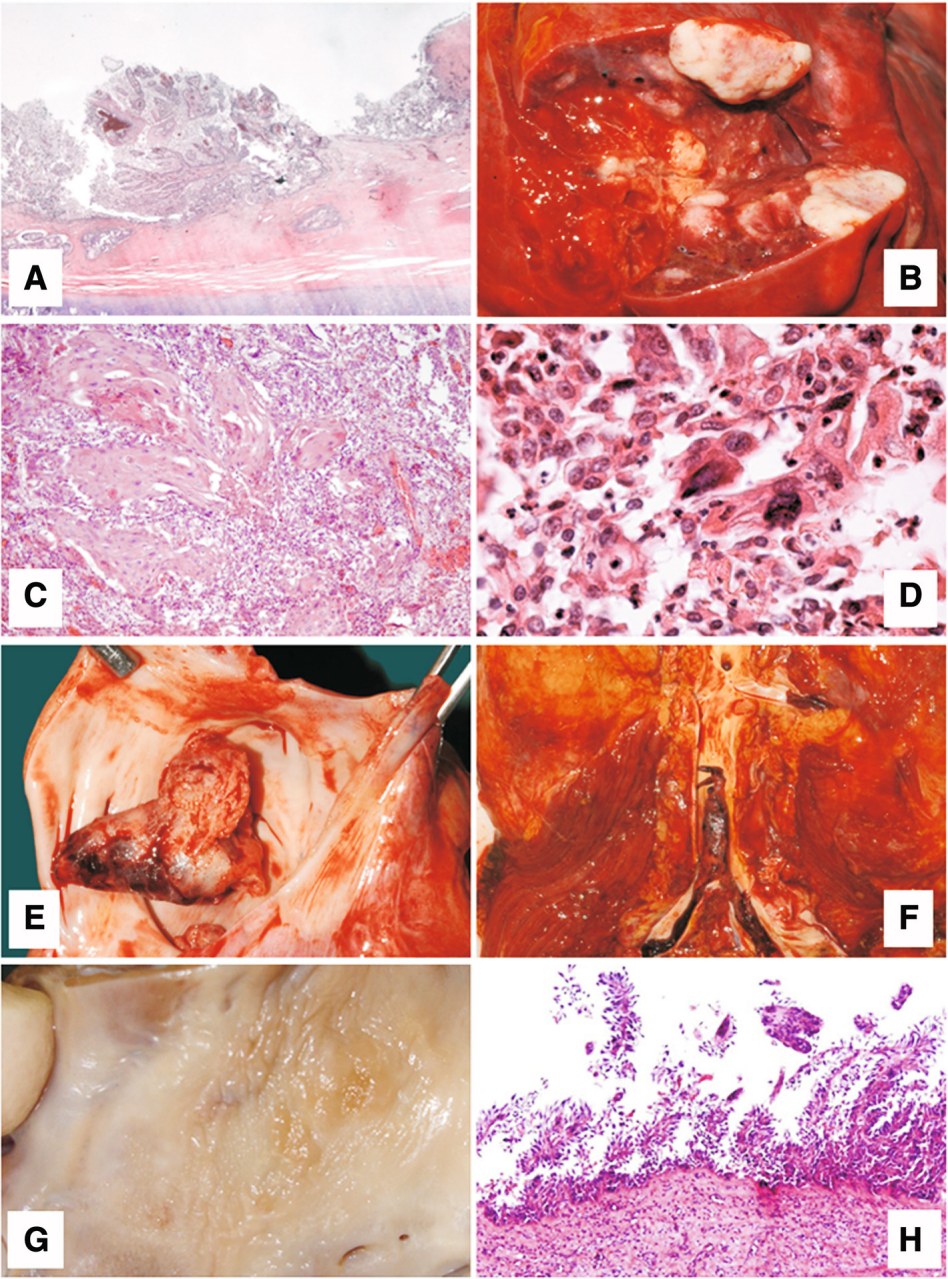
**Post-mortem autopsy results.** Laryngeal respiratory papillomas with focal squamous metaplasia **(A)**; multiple nodules **(B)** of well differentiated squamous cell carcinoma **(C)**; shed tumor cells with marked cytologic atypia, reminiscent of respiratory papilloma **(D)**; left atrial thrombus **(E)** and thromboembolus riding on the aorto-iliacal bifurcation **(F)**; area of endocardial papillomatosis in the left atrium **(G, H)** as the basis for recurrent thrombosis.

**Figure 2:**
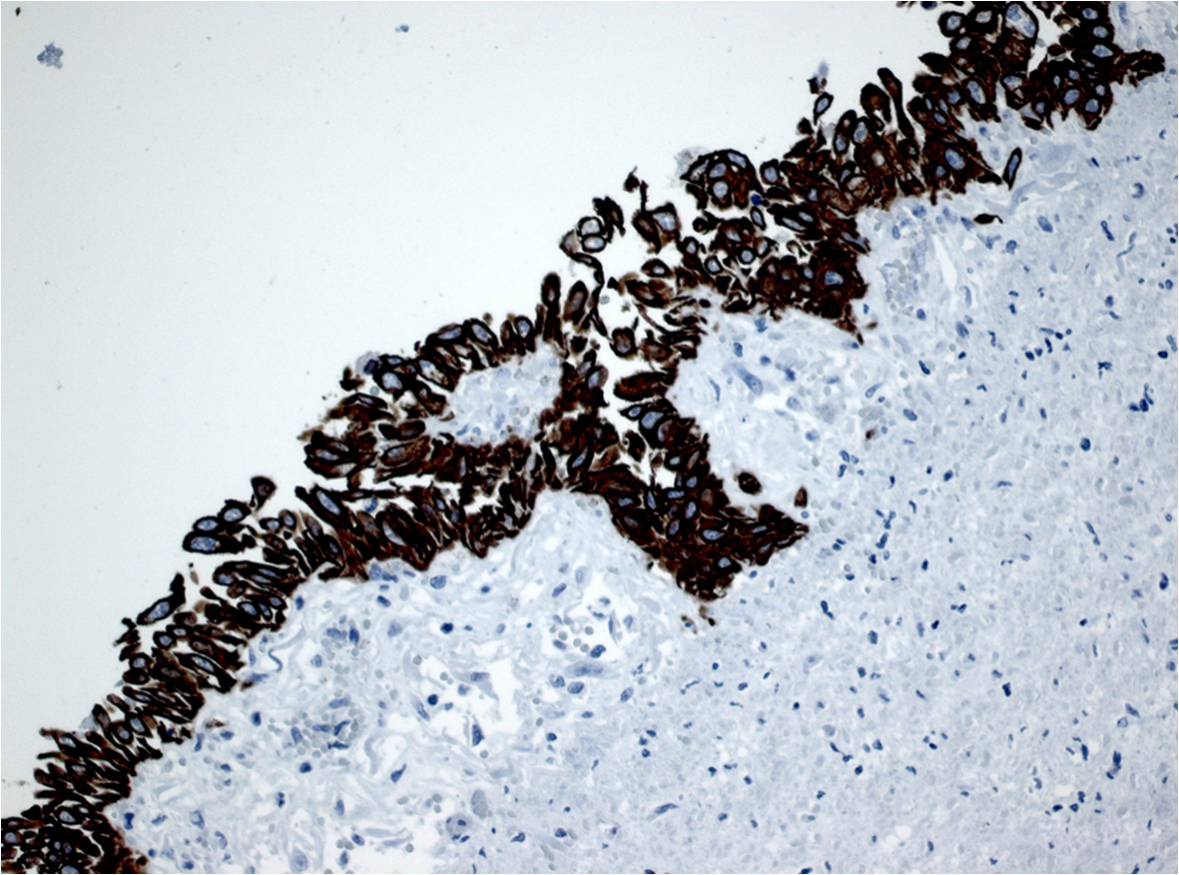
Immunohistochemical staining for the expression of cytokeratin 5/6, a typical marker of squamous epithelium, on a section of a focal squamous papillary metaplasia of the left atrium.

Post mortem examinations revealed that the patient presented with a papillomatous neoplasia of the trachea and main bronchi with what appeared to be microinvasive growth (Figure 1A). Canalicular dissemination within the respiratory tree had occurred. Notably, all pulmonary lobes were affected by multiple confluent well-differentiated squamous cell carcinomas (Figure 1B) with minor foci of keratinisation (Figure 1C) and focal necrosis, developing on the basis of respiratory papillomas with marked cytological atypias (Figure 1D). In the left atrium, a large thrombus was found (Figure 1E, F) behind a sessile papillomatous lesion (Figure 1G), structurally, cytologically, and immunohistochemically identical to the tracheal and pulmonary respiratory papillomas (Figure 1H). Immunohistochemical examination revealed cytokeratin 5 and 6 expression in all neoplastic lesions indicating that the lesion was of epithelial origin (Figure 2). Notably, no lymph node or hematogenous metastases were found.

Molecular biological analyses revealed the presence of HPV DNA in autoptic tissues of lung tumors and left atrial papillomas. In addition, HPV DNA was detected in the left atrial thrombus and the aorto-iliac thromboembolus. HPV-genotyping by nested multiplex PCR [14] revealed strong bands specific for HPV6/11. In order to distinguish between HPV6 and HPV11 DNA, direct sequencing was performed (see Additional file 1) demonstrating the exclusive presence of HPV11 in lung tissue and atrial papillomas which was confirmed by LiPa V2 genotyping as an independent method of HPV detection.

## Conclusion

JORRP is a rare disease which is typically diagnosed in early childhood. It is thought to be caused by the HPV types 6 and 11, which may have been vertically transmitted to the child at birth. It usually affects the larynx, but up to 5% of the patients show involvement of trachea and bronchi and 1% of patients develop manifestations within the lung parenchyma [1,2]. Rarely spontaneous carcinomatous transformation occurs [3], but distant metastases do not develop [2]. We here describe a 19-year-old male patient with an aggressive form of JORRP that required numerous interventions after diagnosis. Scattered papillomas were found in the trachea, bronchi, and both lungs. In multiple locations, bilateral malignant transformation into well-differentiated squamous cell carcinomas had occurred. HPV11 was detected in laryngeal biopsies at high viral load, suggestive of an on-going productive infection. Moreover, HPV11 was detected in autopsy material of the pulmonary squamous cell carcinomas, the left atrial papilloma, the left atrial thrombus, and the thromboembolic material. The same HPV type was isolated and cloned from the laryngeal biopsy. Sequencing of the patient’s HPV11 isolate showed that it is 99% identical to the HPV11 reference nucleotide sequence [12]. It revealed 15 mutations previously observed in an HPV11 isolate from a squamous cell carcinoma of the penis [15,16].

HPV infections usually remain localised and viral particles are not shed into the blood stream. Interestingly however, high viral genome copy numbers were detected in the plasma fraction of the patient’s blood, but not in the cellular fraction demonstrating that the viral genomes were not cell associated. The increased number of viral genomes detected within the plasma as compared to the whole blood (Table 2) is explained by the exclusion of the HPV-negative cellular fraction, which ultimately led to a concentration of the remaining plasma fraction. In connection with the high viral load within the serum, our patient presented high antibody titres against the viral structural protein L1. The antibody titre measured in the 2007 sample (Table 2) is one of the highest ever seen in our laboratory, which usually processes samples from patients vaccinated with the quadrivalent vaccine against HPV6, 11, 16 and 18. Interestingly, no antibodies against the oncoproteins E6 and E7 were detected. A similar immune response against the structural proteins of the virus is typically induced by HPV vaccines or by viral particles entering the blood stream, however, we were not able to show the presence of viral particles within the patient’s plasma. As HPV infections usually remain localised and viral particles are not shed into the blood stream, we hypothesise that a potential angioinvasion of the lung squamous cell carcinoma may have provided a way which enabled viral DNA to enter the blood stream. On another note, it is possible that the high HPV DNA levels originate from necrotic cells or thrombi shed from the endocardial lesion and/or respiratory tumors. In a study from Maloney *et al*., only 20% of the patients show detectable antibody levels [17]. Interestingly, those three reported patients also had the highest levels of HPV11 viral load and the highest average numbers of annual surgical procedures. HPV DNA has previously been detected within blood cells of healthy individuals [18] and in the plasma of HIV-1 patients [19] and women with cervical cancer [20,21]. However, none of these studies examined the immune response.

From the history of our patient it appears that the spread of the HPV infection was not contained by high antibody titres directed against L1. Alternatively, spread of infection might have occurred at a very early stage especially considering that multiple surgical interventions might have increased the risk for tracheal and pulmonary involvement. Viral DNA could therefore have persisted for a considerable time before disease progression and we speculate that a high antibody titre might reflect an increase in disease progression and an elevation of productive infection within existing papillomas.Post mortem examination revealed that descending tracheobronchial respiratory papillomas had undergone squamous metaplasia and subsequent malignant transformation into well-differentiated squamous cell carcinomas. Based on the structural and cytological similarity, including cytological atypia of the tracheobronchial, pulmonary and atrial papillomatous lesions, we conclude that all, especially the latter lesion, were HPV-induced. More importantly, these papillomatous lesions were the source for the recurrent thromboembolic events (Figure 1F) that had led to two episodes of Leriche’s syndrome and to multiple ischemic infractions in various organs, including spleen, kidneys, lower extremities, brain, and heart.

On the basis of the detection of HPV11 DNA in the atrial papilloma, we demonstrated the presence of a very rare endocardial papilloma [22]. To our knowledge this is the first report of an HPV-induced endocardial papilloma as the source of fatal thromboembolic complications during the course of canalicular disseminating HPV11-associated longstanding JORRP disease with malignant transformation into well-differentiated squamous cell carcinoma. In addition, this case is remarkable considering the high levels of viral DNA detected within the patient’s serum and the high immune response directed against the viral structural surface protein L1. A humoral immune response against structural proteins is outmost uncommon in RRP patients and may in our case be explained by the presence of productive papilloma tissue. In summary, we here reported a severe case of JORRP hallmarked by HPV11 DNAemia and very high L1-antibody titres. Furthermore, our unusual and unexpected finding of the extent of malignant transformation and the discovery of a very rare fatal endocardial lesion highlight the unpredictability of JORRP and the complexity of its clinical management.

### Ethics and consent

Ethical approval for the Cidofovir inhalation therapy was obtained from the Ethics Committee of the University Hospital Tuebingen. Written informed consent for the inhalation therapy and the publication of this case report and accompanying images and data was obtained from the patient and his next of kin. A copy of the written consent is available for review by the Editor of this journal. All methodology reported in this paper served for the sole purpose of diagnostics.

## Competing interests

The authors declare that they have no competing interests.

## Authors’ contributions

PSM was the leading clinician in this case, who was involved in acquisition and interpretation of clinical data and contributed in the writing of the article. MZ drafted the manuscript, contributed to the study design, was involved in acquisition, analysis and interpretation of molecular-biological data. RK, MH, JT were involved in the acquisition of clinical data. MP, AI acquired molecular-biological data. FS substantially contributed to the manuscript. KS was the leading pathologist and involved in acquisition of the autopsy data. TI was the leading molecular biologist, substantially contributed to the writing of the article. All authors have read and approved the final manuscript.

## Supplementary Material

Additional file 1 Methods. Click here for file
