# Supplementary material for: A case of recurrent respiratory papillomatosis with malignant transformation, HPV11 DNAemia, high L1 antibody titre and a fatal papillary endocardial lesion
Source: Virol J. 2014 Jun 18;11:114. doi: 10.1186/1743-422X-11-114 (PMC4076064; doi:10.1186/1743-422X-11-114)
Supplement: Additional file 1 — Methods. [file 1743-422X-11-114-S1.pdf]

Additional File 1: Methods

Molecular analyses. Fresh laryngeal biopsies, blood samples as well as paraffin-embedded (FFPE) tissue from laryngeal papillomas, a thrombo-embolectomy specimen and autoptic tissues of the lungs, left atrium including papillomas, thrombotic/thromboembolic material and the aorto-iliac bifurcation were investigated by different methods.
DNA was extracted using either the MagNA Pure compact nucleic acid purification system (Roche, Mannheim, Germany), the Qiagen EZ1 DNA tissue kit or, in the case of FFPE material, the Qiagen QIAmp Blood mini kit (Qiagen, Hilden, Germany). HPV genotyping was carried out using the INNO-LiPA v2 HPV genotyping kit (Innogenetics, Gent, Belgium) or by a method described by Sotlar *et al*. for the FFPE material [1].

Benzonase treatment. Benzonase treatment was performed following the manufacturer's instructions. Dilutions of HPV16-positive SiHa cell DNA were used as a control. Treatment was performed on dilutions of plasma from two different time points. Detection of viral DNA was performed by INNO-LiPA HPV Genotyping Extra.

Cloning and sequencing of HPV11. The full length genome of HPV11 was amplified, from total DNA obtained from a laryngeal biopsy, using the TempliPhi, φ29 DNA Polymerase Based Rolling Circle Amplification kit (GE Healthcare, Freiburg, Germany), cloned into pDrive (Qiagen, Hilden, Germany) and was completely sequenced based on overlapping PCR fragments.

Quantitative real-time PCR. 5µl of eluted DNA was subjected to q-PCR using the quantitative PCR mastermix for SYBR Green I reagents (Eurogentec, Seraing, Belgium) and HPV11-specific primers to determine the viral genome copy number. Primers matching the human β-globin gene were used to normalize HPV11 copy numbers in relation to the total number of human cells. Primers used for HPV11 copy number detection were as follows: F-5' CGTACGAGCCCTGTATTGGT-3' and R-5'-TTAAAAAGGCCCTTGCATTG-3'; β-globin F-5'-GGTTGGCCAATCTACTCCCAGG-3'and R-5'-GCTCACTCAGTGTGGCAAAG-3'.

HPV antibody detection. Serum antibodies against HPV L1 major capsid proteins of a broad variety of HPV types as well as E6 and E7 of HPV11 were analysed by multiplex serology using bacterially expressed viral proteins fused to glutathione S-transferase as antigens as described previously [2, 3].

Immunohistochemistry. Cytokeratin 5/6 expression was detected using a primary anti-mouse antibody (M7237, DAKO).

References

1. Sotlar K, Diemer D, Dethleffs A, Hack Y, Stubner A, Vollmer N, Menton S, Menton M, Dietz K, Wallwiener D *et al*: **Detection and typing of human papillomavirus by e6 nested multiplex PCR**. *Journal of clinical microbiology* 2004, **42**(7):3176-3184.
2. Michael KM, Waterboer T, Sehr P, Rother A, Reidel U, Boeing H, Bravo IG, Schlehofer J, Gartner BC, Pawlita M: **Seroprevalence of 34 human papillomavirus types in the German general population**. *PLoS pathogens* 2008, **4**(6):e1000091.

3. Waterboer T, Sehr P, Michael KM, Franceschi S, Nieland JD, Joos TO, Templin MF, Pawlita M: **Multiplex human papillomavirus serology based on in situ-purified glutathione s-transferase fusion proteins**. *Clin Chem* 2005, **51**(10):1845-1853.
